# Transplacental Chikungunya Virus Antibody Kinetics, Thailand

**DOI:** 10.3201/eid1211.051560

**Published:** 2006-11

**Authors:** Veerachai Watanaveeradej, Timothy P. Endy, Sriluck Simasathien, Angkool Kerdpanich, Napuschon Polprasert, Chanchai Aree, David W. Vaughn, Ananda Nisalak

**Affiliations:** * Phramongkutklao Hospital, Bangkok, Thailand;; † Walter Reed Army Institute of Research, Silver Spring, Maryland, USA;; ‡ US Army Medical Research and Materiel Command, Fort Detrick, Maryland, USA;; § Armed Forces Research Institute of Medical Sciences, Bangkok, Thailand

**Keywords:** dengue, chikungunya, alphavirus, Aedes, transplacental, antibody, Thailand, kinetics, dispatch

## Abstract

Antibodies to chikungunya virus were detected by hemagglutination-inhibition assay in 33.6% of 2,000 infants' cord sera at delivery. Follow-up of 24 seropositive infants showed that the half-life of antibody persistence was 35.5 days. Chikungunya virus infection is common in Thailand, and routine use of diagnostic assays is needed.

Chikungunya virus (CHIKV, family Togaviridae, genus Alphavirus) was first isolated during an epidemic in Tanzania in 1952 and 1953 (*1*). CHIKV disease can manifest as a syndrome involving fever, rash, and arthralgia syndrome (*2*) and can produce clinical signs and symptoms that are difficult to distinguish from those of dengue fever or dengue hemorrhagic fever. CHIKV and dengue virus (DENV) are both transmitted by Aedes mosquitoes, such as A. aegypti and A. albopictus. Thus, many risk factors for CHIKV and DENV infections are similar. The diagnosis of dengue in Thailand is made primarily by clinical symptoms and a complete blood count according to World Health Organization guidelines. However, the major clinical features of dengue overlap with those of other causes of febrile illnesses (*3*). In addition, denguelike illness has occasionally been reported in patients without evidence of anti-dengue antibody seroconversion (*4**,**5*).

The objectives of this study were to assess the seroprevalence of antibodies to CHIKV in a sample of pregnant women and the kinetics of transplacentally transferred antibodies to CHIKV. This is the first study of serologic features of CHIKV in a large Thai sample. We also examined antibodies to dengue viruses in the same sample (*6*) to increase our understanding of the epidemiologic features of both diseases.

## The Study

Two thousand pregnant women with uncomplicated pregnancies at the time of delivery at the Phramongkutklao Hospital from March 1998 through October 1999 gave informed consent to participate in this study. Antibody titers to CHIKV were measured by hemagglutination-inhibition (HI) assay in all 2,000 cord serum samples. Antibodies in cord blood are transferred from the mother and can reflect previous infection. A subset of 250 mothers and their infants were enrolled to compare the rate of transfer of maternal antibodies. Within this subset, 101 infants had serial serum sampling at 1, 2, 4, 6, 9, 12, 15, and 18 months of age.

HI titers to CHIKV and DENV were determined at the Armed Forces Research Institute of Medical Sciences, Bangkok, Thailand. Assays were performed according to the method of Clarke and Casals, modified for the microtiter system for each virus as previously described (*6**,**7*). HI titers >10 were considered positive. CHIKV is the only alphavirus known to circulate in Thailand; antibodies to other alphaviruses were not expected in this study, nor were they assayed. However, Ross River virus, Getah virus, Sindbis virus, and Bebaru virus have been reported to circulate in countries that border Thailand (*1*).

**Figure 1:**
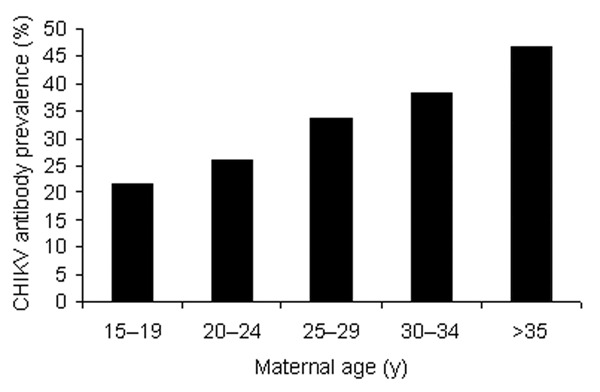
Age-specific seroprevalence of maternal antibody to chikungunya virus (CHIKV) measured by hemagglutination-inhibition assay in infant cord blood at the time of delivery.

The mean age of the 2,000 mothers was 26.4 years (range 15–45 years). Most volunteers (79.9%) lived in Bangkok. Of these, 672 (33.6%) and 1,937 (96.9%) were seropositive for CHIKV and DENV, respectively. The seroprevalence of antibodies to CHIKV increased with age (Figure 1), and ≈47% of mothers >35 years of age were seropositive to CHIKV. The degree of CHIKV-specific antibodies transferred to infants was determined in 250 randomly selected mother-infant pairs. Of 250 mothers, 79 (31.6%) were seropositive for CHIKV, and 64 (81.0%) of these mothers transferred antibodies to their babies. We compared HI titers between mothers and cord sera; 58% had the same titers, 31% of cord sera had higher titers, and 11% of cord sera had lower titers. This finding was consistent with an active transport mechanism across the placenta. Similar findings were reported for DENV-specific antibodies (*6**,**8*). Fifteen (19%) infants born to seropositive mothers did not have detectable titers of antibodies to CHIKV.

**Figure 2:**
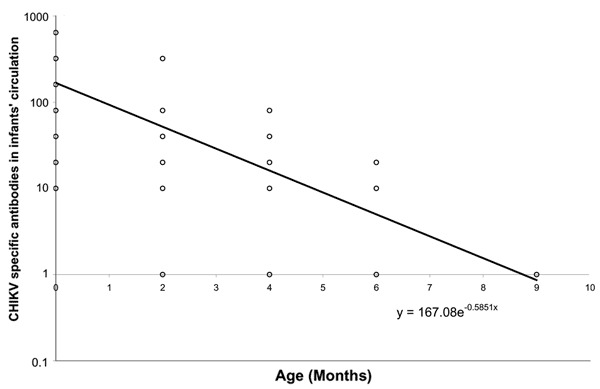
Half-life of maternal antibody to chikungunya virus (CHIKV). Each dot represents the titer at that given age; >1 participant can share the same dot. CHIKV hemagglutination-inhibition titers in infants' sera are plotted on a logarithmic scale, with the line of best fit calculated by exponential regression on a linear scale.

Of the 79 mothers who were seropositive to CHIKV, 28 agreed to further follow-up study; their infants were followed up until 18 months of age. Four infants were negative on cord blood testing and remained negative until 18 months of age. Of 24 infants whose cord blood was positive, 8.3%, 33.3%, 87.5%, and 100% lost their antibodies to CHIKV by 2, 4, 6, and 9 months of age, respectively. The half-life of antibody to CHIKV was calculated by plotting the antibody titer versus age to 18 months on both linear and logarithmic scales. Using SPSS software (SPSS Inc., Chicago, IL, USA), we calculated the line of best fit by exponential regression (Figure 2). From this curve, we calculated the half-life of maternal antibodies to CHIKV in infants to be 35.5 days.

## Conclusions

In Thailand, after the first reported cases of CHIKV infection were confirmed by serologic analysis in 1960 (*9*), a serosurvey was conducted in 1976 in a rural population with an overall antibody prevalence of 24.6% that increased with age (*10*). The higher prevalence seen in this study (33.6%) might be explained by a larger sample that is more capable of sustaining transmission and increased vectors. A nationwide serologic study of both DENV and CHIKV in Thai patients took place in 1974–1976. This study suggested that CHIKV was not a substantial health problem (*11*). Disease caused by CHIKV, unlike DENV, is not a reportable disease, and laboratory diagnosis is not routinely available. As a result, it is probably underreported, which would reduce physicians' index of suspicion, resulting in further underreporting. However, epidemics of CHIKV disease in Thailand have been periodically documented.

The clinical-to-subclinical ratio was 1:1 to 1:6 for DENV infection (*12*) and was ≈1.8:1 for CHIKV infection (C.G. Beckett, pers. comm.). This report shows the reemergence of CHIKV infection in Thailand; the ratio of seroprevalence of DENV infection to CHIKV infection was 2.9:1. National surveillance reported 130,000 dengue illnesses in 1998 (*13*). If the ratio is applied, we can estimate >44,000 persons infected with CHIKV in the same year. Therefore, infection with this virus may be more common than is believed. In a previous study of Thai children hospitalized with presumptive dengue hemorrhagic fever, ≈20% of diagnoses were ultimately changed to acute CHIKV infection (*4*).

Antibodies to CHIKV in infants' circulation may protect them from the illness until 9 months of age. This finding is consistent with the observation that denguelike illness is rarely seen in infants. A vaccine for CHIKV is still in early stages of development (*14*); the 9-month persistence of antibodies to CHIKV provides insight to the optimal age of vaccination, should a vaccine become available. This study shows substantial CHIKV circulation in a DENV-endemic country. Since signs and symptoms of the disease are similar, surveillance for viruses and the diseases they cause should be developed and maintained.
